# Genetic Analysis of Neuroligin 4Y Gene in Autism Population of India

**DOI:** 10.1055/s-0041-1736236

**Published:** 2021-09-28

**Authors:** Rajat Hegde, Smita Hegde, Suyamindra S. Kulkarni, Aditya Pandurangi, Pramod B. Gai, Kusal K. Das

**Affiliations:** 1 Laboratory of Vascular Physiology and Medicine, Department of Physiology, Shri B.M. Patil Medical College, Hospital and Research Centre, BLDE (Deemed to be University), Vijayapura, Karnataka, India; 2 Karnataka Institute for DNA Research, Dharwad, Karnataka, India; 3 Human Genetics Laboratory, Department of Anatomy, Shri B.M. Patil Medical College, Hospital and Research Centre, BLDE (Deemed to be University), Vijayapura, Karnataka, India; 4 Department of Psychiatry, Dharwad Institute of Mental Health and Neurosciences, Dharwad, Karnataka, India

**Keywords:** autism, neuroligin 4Y, India, novel missense mutation, male predominance

## Abstract

**Background** Autism is one of the most complex, heterogeneous neurological disorders. It is characterized mainly by abnormal communication, impaired social interaction, and restricted behaviors. Prevalence of autism is not clear in Indian population.

**Aim** The present study hypothesized that Y chromosome plays role in sex bias of autism in Indian autistic population. To investigate our hypothesis, we underwent genetic analysis of neuroligin 4Y [ *NLGN4Y* ] gene by sequencing 85 male autistic children after screening large population of 1,870 mentally ill children from North Karnataka region of India.

**Result** Detailed sequencing of the single targeted gene revealed nine variants including, one novel missense mutation and eight synonymous variants; this accounts for 88.9% of synonymous variants. A single novel missense mutation is predicted to be nonpathogenic on the functions of neuroligin4Y protein but it slightly affects the local configuration by altering the original structure of a protein by changing charge and size of amino acid.

**Conclusion** Probably *NLGN4Y* gene may not be the risk factor for autism in male children in Indian autistic population. Functional analysis was an important limitation of our study. Therefore, detailed functional analysis is necessary to determine the exact role of novel missense mutation of neuroligin 4Y [ *NLGN4Y* ] gene especially in the male predominance of autism in Indian autistic population.

## Introduction

Autism [ASD] [MIM 299850] is a heterogeneous neurodevelopmental disorder. Autism is not characterized based on a single symptom. It is usually characterized by the triad of symptoms viz lack of social interaction, abnormal verbal and nonverbal communications and stereotyped or repetitive behaviors. 1 Autism is classified as syndromic and non-syndromic autism. Syndromic autism is one in which patients who have pre-existing neurological disorders, example, a subset of patients with fragile x syndrome, tubers sclerosis, Rett syndrome displays phenotypes which are attributed to ASD. Non-syndromic autism accounts for autism cases that are not linked to any neurological disorders. 2 Neuroligin is trans-synaptic cell adhesion molecule present post-synaptically and plays a very important role in synaptogenesis with presynaptic neurexin. 3 Humans have neuroligin 4X [ *NLGN4X* ] on the X chromosome and neuroligin 4Y [ *NLGN4Y* ] on the Y chromosome. *NLGN4X* and *NLGN4Y* genes share 97% sequence identify. 4 The male bias seen from *NLGN4X* mutations is unclear since *NLGN4Y* plays a function similar to *NLGN4X* and should be sufficient to reimburse for *NLGN4X* ASD-related mutations. This lack of compensation in males suggested that *NLGN4Y* may have an uncharacterized distinct function that needs to be explored. Several studies are reported that synaptic cell adhesion molecules have been strongly involved in autism. Neuroligin has an important role in the maturation and functions of synapses. 5 6 The mechanism of Y chromosome contribution on to neurodevelopmental disorders is still not known very well. Originally, it was thought that Y chromosome contains only a few genes that are primarily involved in sex determination and testicular functions but now it is known to contain numerous genes with diverse functions. 7 Several shreds of evidence strongly suggested that *NLGN4X* deficiencies can cause autism and still there is no clear understanding of sex bias in autism.

We hypothesize that male individuals have both X and Y chromosome so analysis of sequence variants in *NLGN4Y* gene may be associated with sex bias in male autistic individuals. To address this objective, we sequenced all the exonic regions of *NLGN4Y* gene in 85 male autistic children from north Karnataka region of India.

## Methods

### Sample Collection

One-hundred fifty autistic children were identified after screening a large mentally ill population of 1,870 children from the entire North Karnataka region of India ( *n*_male_ = 117, *n*_female_ = 33 mean age = ± 11.5]. Eighty-five male autistic children were included for the genetic analysis of NLGN4Y gene. Screening of autistic children was performed using Diagnostic and Statistical Manual of Mental Disorders-V (DSM-V, American Psychiatric Association, 2000) ( https://www.psychiatry.org/psychiatrists/practice/dsm ) and/or International Classification of Diseases-10 (ICD-10, WHO) ( https://www.who.int/ classifications/ icd/ icdonlineversions/en/). Child with associated neurological disorders and other comorbid diseases was excluded from the study. Around 1 to 2 mL of peripheral blood was collected in EDTA-coated vacutainers and stored in −20°C until further analysis.

### DNA Isolation and PCR Amplification

Genomic DNA was isolated from peripheral blood using blood and tissue DNA isolation kit (QIAGEN, Germany) as per manufactures guidelines. Quality and quantity of isolated genomic DNA were checked using agarose gel electrophoresis and nanodrop UV spectrophotometer (Quawell, Q3000 UV spectrophotometer). Amplification of *NLGN4Y* gene was carried with designed primers using standard PCR reagents (New England Bio Labs, United States). Quality and quantity of PCR product were analyzed.

### Sequencing

PCR products of *NLGN4Y* gene was sequenced using Sanger sequencing kit v3.1 on ABI 3500 Sanger sequencer platform. Sequence data were analyzed with ABI sequence analysis Software v5.4 (Applied Biosystem, United States).

### Bioinformatics Analysis

Pathogenic effect of missense mutation was analyzed using Insilco tools like PROVEAN, SNAP2, polyphen2, SNP&GO, and CADD. Conservation status of amino acid residues of NLGN4Y protein at 163 position was checked using the ConSurf Server ( https://consurf.tau.ac.il/ ). 8 Three-dimensional structure of wild type and mutant protein was developed using Swiss-model and structures were visualized and analyzed using UCSF Chimera tool.

## Results

**Fig. 1:**
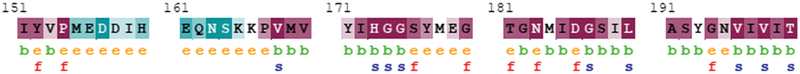
Conservation status of p.N163K mutation, amino acid residue at 163 position is not conserved; it is variable and exposed residue.

Detailed screening of 1,870 mentally ill children below 18 years of age from North Karnataka population of India revealed 150 autistic children [ *n*_male_ = 117, *n*_female_ = 33 mean age = ± 11.5] which accounts for 8.02% of autism in North Karnataka region of India. Sanger Sequence analysis of neuroligin 4Y gene from 85 male autistic children revealed the nine variants, which include one missense and eight synonymous variants. Four variants which were recorded in our study cohort are not previously recorded in any in house human SNP databases viz dbSNP, 1000 genomes, ExAc and ClinVar shown in Table 1 . Novel missense, p.N163K mutation was recorded in three autistic children and clinical features of those autistic children with missense mutation are shown in Table 2 . Pathogenicity prediction of missense variants was analyzed using Insilco tools viz PROVEAN, POLYPHEN2, SNAP2, SNP&GO, and CADD. Only Missense variant, p.N163K was found to be harmless on the functions of *NLGN4Y* protein by PROVEAN, POLYPHEN2, SNAP2, SNP&GO, and CADD shown in Table 3 . Conservation status analysis of NLGN4Y protein sequences shows that amino acid residue at 163 position is not conserved; it is variable and exposed residue according to the neural network algorithm shown in Fig. 1 .

**Table 1:** Showing list of mutations recorded in our study cohort

Variation	N. change	A.A Change	SNP id	Frequency of mutation
Missense	g. 205526 C > A	p.N163K	Not recorded	3 (3.5%)
Synonymous	g.312652 T > C	p. H447=	rs777234513	4 homozygous (4.7%) 2 heterozygous (2.3%)
Synonymous	g.312781C > T	p.G490=	rs767447455	3 (3.5%)
Synonymous	g.312787 A > G	p.E492=	rs750273940	3 homozygous (3.5%) 2 heterozygous (2.3%)
Synonymous	g.312826 A > C	p.T505=	Not recorded	1 (1.2%)
Synonymous	g.312844 T > C	p.N512=	Not recorded	2 (2.3%)
Synonymous	g. 312847 C > T	p.F513=	Not recorded	1 (1.2%)
Synonymous	g. 312871 T > C	p.S520=	rs1423308667	1 (1.2%)
Synonymous	g. 312880 G > C	p.V523=	rs753006927	3 (3.5%)

**Table 2:** Clinical features of autism children with missense mutation, p.N163K of NLGN4Y gene

Demographic character	Child 1	Child 2	Child 3
Ethnic origin	Indian	Indian	Indian
Sex	Male	Male	Male
Age of father at child's birth	38	29	35
Age of mother at child's birth	33	19	34
Consanguineous marriage	No	Yes	No
Prenatal damage	preeclampsia	None	None
Postnatal damage	None	None	Birth asphyxia
IQ	25	30	28
CARS Score and Severity	40; Severe	44; Severe	52 ; Severe
Comorbidity	None	None	None

**Table 3:** Showing pathogenicity prediction of a missense variant

Variant	PROVEAN	SNP&GO	PolyPhen2	SNAP2	CADD score
p.N163K	Neutral Score: −0.992	Neutral 0.381	Benign Score: 0.023	Neutral Score: −79	19.34 [Raw score 2.010689]

**Fig. 2:**
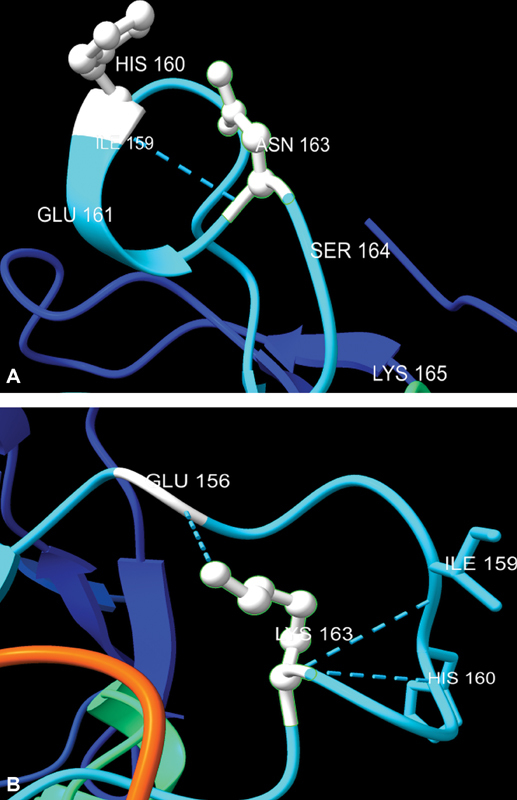
( **A** ) Three-dimensional model of wild type protein; wild type amino acid residue is small and neutrally charged. ( **B** ) Three-dimensional model of mutant, p.N163K protein. Mutant residue is bigger than wild type residue and it is positively charged.

Three-dimensional protein modeling analysis of NLGN 4Y protein revealed that mutant residue is bigger than wild type residue and it possesses a positive charge whereas wild type protein possesses neutral charge. The wild type of residue is predicted to be located in its preferred secondary structure, a turn but the mutant residue prefers to be in another secondary structure; therefore, the local conformation will be slightly destabilized shown in Fig. 2A and Fig. 2B . The mutated residue is located in a domain that is important for binding of other molecules. Mutation of the residue might disturb this function of NLGN4Y protein.

## Discussion

Autism [MIM 299850] is a complex neurological condition which is characterized by abnormal social interaction, verbal and nonverbal communication and impaired behaviors. “Autism,” the term was first used by Ukrainian-Austrian-American psychiatrist, Leo Kenner in 1943. 9 Rates of autism cases are increasing globally over the period of time when it comes to the Indian perspectives, cases are increasing dramatically, and it may be due to increased scientific knowledge and awareness or it may be an improper diagnosis. In recent days, neuroligin gene is the most targeted gene for the molecular studies on neurological disorders like autism, anxiety, attention deficit hyperactivity disorder and intellectual disability due to its active role in synaptogenesis. 10 11 12 13

The Simons Foundation Autism Research Initiative (SFARI) [geneSFARI.org] lists four genes of Y chromosome associated with autism viz *NLGN4Y, ASMT, USP9Y,* and *SHOX* . 14 Only a few studies have been undertaken till now to study the role of *NLGN4Y* gene in autism. Studies conducted in 2005 and 2006, failed to identify the variants in the *NLGN4Y* gene in autistic patients. 15 16 But later several studies, record polymorphisms of *NLGN4Y* gene involved in neurodevelopmental disorder and synaptic functions have been associated with autism. 17 18 Peripheral blood *NLGN4Y* gene expression showed an increased risk of autism in children with XYY symptoms. 7

In the present study, for the first time in Indian autistic population, we analyzed a cohort of 85 male autistic children under the age of 18 years after screening large neurological disorders population. Our study population records 7.4% of autism over different neurological disorders. Sequencing analysis of the entire exonic region revealed 09 mutations. One is novel missense mutation and reaming 08 (88.9%) are synonymous variants. Out of eight synonymous variants, five (62.5%) were already reported in in-house human SNP databases. Insilco functional effect prediction of a novel missense mutation, p.N163K by PROVEAN, POLYPHEN2, SNAP2, SNP&GO, and CADD shows nonpathogenic effects on the functions of neuroligin 4Y protein. But structural analysis of p.N163K mutant protein shows slight destabilization in the local configuration by positioning on different secondary structure, a turn and it might cause bumps in protein structure due to differences in the size of amino acid. Meanwhile, the mutation introduces a charge; this can cause repulsion of ligands or other residues with the same charge.

Structural and functional prediction of novel missense mutation indicates slight changes related only to the structure of neuroligin 4Y protein, and not to the functions of the protein. Absence of functional analysis of gene was the important limitation of our study.

## Conclusion

Mutation in *NLGN4Y* may be an uncommon cause of autism in Indian autistic population. But further detailed functional investigation of neuroligin 4Y gene in autism is important to understand the male predominance of autism and increased rate of autism in males in India autistic population.
